# Acute Pulmonary Embolism: Prognostic Role of Computed Tomography Pulmonary Angiography (CTPA)

**DOI:** 10.3390/tomography8010042

**Published:** 2022-02-14

**Authors:** Giulia Zantonelli, Diletta Cozzi, Alessandra Bindi, Edoardo Cavigli, Chiara Moroni, Silvia Luvarà, Giulia Grazzini, Ginevra Danti, Vincenza Granata, Vittorio Miele

**Affiliations:** 1 Emergency Radiology, Careggi University Hospital, 50134 Florence, Italy; giulia.zanto@gmail.com (G.Z.); bindi.alessandra@gmail.com (A.B.); edoardocavigli@yahoo.it (E.C.); chiaramoroni73@gmail.com (C.M.); luvaras@aou-careggi.toscana.it (S.L.); grazzini.giulia@gmail.com (G.G.); ginevra.danti@gmail.com (G.D.); vmiele@sirm.org (V.M.); 2 Italian Society of Medical and Interventional Radiology (SIRM), SIRM Foundation, 20122 Milan, Italy; 3 Radiology Division, Istituto Nazionale Tumori IRCCS Fondazione Pascale, 80131 Naples, Italy; v.granata@istitutotumori.na.it

**Keywords:** pulmonary embolism, CTPA, vascular, emergency

## Abstract

Computed Tomography Pulmonary Angiography (CTPA) is considered the gold standard diagnostic technique in patients with suspected acute pulmonary embolism in emergency departments. Several studies have been conducted on the predictive value of CTPA on the outcomes of pulmonary embolism (PE). The purpose of this article is to provide an updated review of the literature reporting imaging parameters and quantitative CT scores to predict the severity of PE.

## 1. Introduction

Pulmonary embolism (PE) is defined by embolic occlusion of the pulmonary arterial system. PE is the third most frequently occurring cause of cardiovascular death after stroke and myocardial infarction, causing ≥ 300,000 deaths per year in the US [1,2]. Right ventricular dysfunction (RVD), defined as a rapidly progressive congestion syndrome resulting from impaired filling and/or reduced flow out of the right ventricle, triggered by acute pressure overload, is the leading cause of death in severe PE [3]. Signs of RVD and hemodynamic instability, such as tachycardia, low systolic blood pressure, respiratory failure, and syncope, are associated with a poor prognosis and a high risk of early mortality (within 30 days) [3]. Another, albeit late but potentially fatal, consequence of PE is the development of chronic pulmonary hypertension.

Among imaging tests, Computed Tomography Pulmonary Angiography (CTPA) is considered the first-line diagnostic technique in patients with suspected PE with sensitivity and specificity values between 96 and 100% and between 89 and 98%, respectively [4]. In the European Society of Cardiology guidelines, CTPA is indicated as Class IC in patients with high suspicion of PE, even if hemodynamically unstable [5]. Moreover, if the CTPA is regular in patients with low or intermediate clinical probability, the diagnosis of PE can be ruled out without further testing (Class IA) [5]. On the other hand, over-testing for PE remains a major health problem, especially in University hospitals [6,7]. In fact, it is estimated that 9.4% of CTPA could be avoided [8]. In addition, several studies have been performed on the potential role of CTPA as an ancillary tool in establishing patient prognosis in PE [9,10,11,12].

This article aims to provide an updated review of the literature reporting imaging parameters and quantitative Computed Tomography (CT) scores useful in predicting PE severities to identify patients who need aggressive therapy or intensive observation.

## 2. Pulmonary Artery Clots Burden Indexes

The degree of clot obstruction within the pulmonary arterial tree can be estimated using four different indexes. The first angiographic scores adapted to spiral CT were those of Walsh and Miller [13]. Subsequently, Qanadli et al. and Mastora et al. proposed two different indexes specifically studied for CTPA to quantitatively assess the severity of acute PE [14,15]. The CT Obstruction Index (CTOI) as described by Qanadli et al. is calculated as the sum of the individual scores per artery divided by 40 (the maximum total score) and converted into a percentage. The unit score per artery is calculated by attributing a unit value to each segmental pulmonary artery with thromboembolism (max 10 for each lung) and multiplying it by a weight factor (1 for partial obstruction or 2 for total obstruction) [14]. Mastora et al. described an index like CTOI, which is also related to some appreciable findings on echocardiography, such as the presence of pulmonary hypertension and cor pulmonale [15]. Currently, the Qanadli index is commonly used to assess the severity of acute pulmonary embolism (APE) at the CTPA. As suggested by the Authors, two groups of patients are identified. The high-risk subgroup has a CTOI ≥ 20 and the low-risk subgroup has a CTOI < 20 [14]. The ability to select high-risk PE patients using pulmonary obstruction indexes was examined by several studies. Faghihi Langroudi et al. investigated the association between CTOI and atrial size in patients with APE, showing that higher clot load is associated with smaller (left atrium) LA size and increased (right atrium) RA/LA ratios [16]. In a small study of 35 patients with PE, Praveen Kumar et al. found that CTOI was a strong independent predictor of RVD in PE, linearly correlating to several variables associated with increased morbidity and mortality, allowing an accurate risk stratification selection of patients who needed more aggressive treatment [17]. Rotzinger et al. recently reported that patients with PE, excluding those with cardiopulmonary comorbidities or pulmonary neoplasms and with CTOI greater than 40%, had significantly higher mortality (*p* < 0.001) than those with CTOI less than 20% [18]. Patients with PE, cardiopulmonary comorbidities, or pulmonary neoplasms had an increased risk of fatal outcomes regardless of CTOI. Nevertheless, the time required for the data analysis would be significantly reduced using computer-assisted techniques such as artificial intelligence, increasing the reliability of vascular obstruction indexes (both CTOI and Mastora), reducing time and costs, and providing additional quantitative parameters for severity disease assessment [19].

## 3. Predictor Findings

### 3.1. RV/LV Ratio

**Figure 1:**
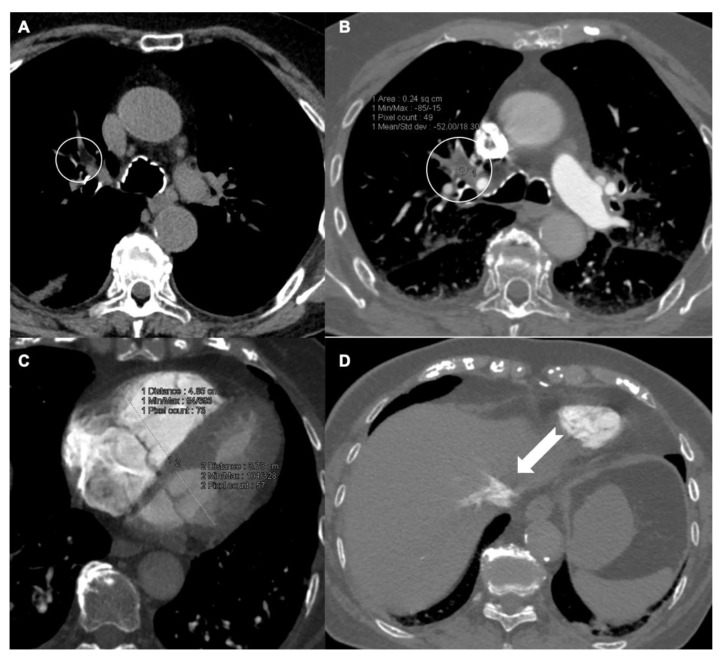
Patient with fat-filling defects in the right upper lobar pulmonary artery in non-contrast (**A**) and contrast (**B**) axial scans. (**C**) CTPA illustration of RV/LV diameter ratio measurement; the measurement in this patient was 1.26. ((**D**), arrow) Reflux of contrast medium extended to hepatic veins (grade III).

As reported by Aribas et al., cardiac chamber diameters should be measured in the short-axis plane. Right ventricle (RV) and left ventricle (LV) diameters are required to calculate the RV/LV ratio [20]. The ratio of RV/LV short axis in CTPA > 1 is a surrogate marker for RV dysfunction because it correlates with the increase in afterload induced by acute embolism on ventricular function. Several studies validated its role as a predictor of short-term mortality (within 30 days) and adverse clinical events in patients with acute PE [9,12,21]. Ayöz et al. described higher troponin levels in patients with RV/LV > 1 [22]. Moreover, in a study by Cho et al., an RV/LV diameter ratio greater than 1 was associated with an increased risk of 2.4 admissions to the intensive care unit [21] (Figure 1).

### 3.2. Diameter of Pulmonary Artery

**Figure 2:**
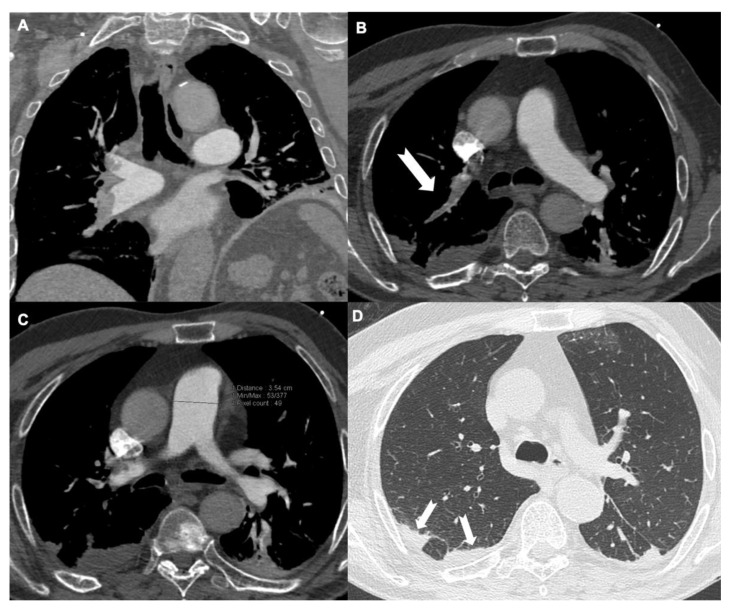
Patient with embolism involving pulmonary arterial branches for the right upper and lower lobe (**A**) and a sub-segmental branch for the right upper lobe ((**B**), arrow). Pulmonary arterial trunk dilatation (35.4 mm) (**C**) and multiple pulmonary infarctions ((**D**), arrows) are shown.

Main pulmonary artery (PA) diameters and the ratio of the pulmonary artery to the ascending aorta have been suggested as indicators of pulmonary hypertension. In a recent study, a significant increase in pulmonary (*p* = 0.003) and aortic (*p* = 0.006) diameter was seen in patients who succumbed to PE compared with those who survived [22]. Furthermore, according to Lyhne et al., an increase in PA diameter led to serious adverse events. In their study, the median PA diameter in patients with adverse outcomes was 29.9 mm (CI 21.8–32.4 mm) (*p* = 0.014) [23] (Figure 2).

### 3.3. Diameter of Coronary Sinus

**Figure 3:**
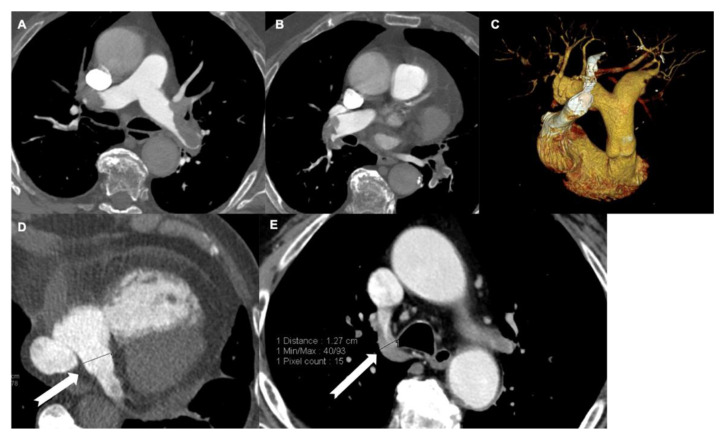
Increase in the diameter of the veins and embolism. A middle-aged man with huge eccentric emboli attached to the vascular wall of both pulmonary arteries (**A**–**C**). (**D**) Dilatation of the coronary sinus (arrow); the measurement in this patient was 21 mm. Dilatation of the azygos vein (12.7 mm) (**E**).

Recently, Cozzi et al. showed that coronary sinus dilatation (>9 mm) is related to an increased risk of all-cause death within 30 days (*p* < 0.05). Compared to the RV/LV ratio, this is poorly affected by changes in the cardiac cycle, contractile motion artifacts, or heart diseases. Therefore, the use of this parameter can provide additional results in predicting outcomes [9] (Figure 3).

### 3.4. Inferior Vena Cava Reflux

Contrast reflux into the inferior vena cava (IVC) is secondary to right heart failure. The degree of reflux can be quantified on a three-point scale where *grade I* does not indicate reflux in the IVC, *grade II* suggests subcardial reflux in IVC, and *grade III* identifies intrahepatic reflux in IVC [24,25]. Several studies showed a positive correlation between IVC contrast reflux and poor prognosis [22,26]. In particular, in the study by Ayöz et al., IVC reflux was present in 81.3% (*p* = 0.001) of patients who succumbed. Furthermore, patients with an RV/LV ratio ≥ 1 had IVC reflux more frequently (*p* = 0.025) than patients with an RV/LV ratio < 1 [22].

### 3.5. Displacement of the Interventricular Septum

The interventricular septum normally bends towards the RV; however, due to the increase in pressure in the right heart sections, it can move towards the LV. Ventricular septum bowing has excellent specificity (100%) but poor sensitivity (26%) in predicting RV dysfunction [27]. However, in the study by Shayganfar et al., abnormal septal morphology was significantly more common among patients with high-risk PE (*p* < 0.01) [10]. The abnormal septal placement is a negative predictor in both CTPA and echocardiography, albeit not consistently. For instance, in a meta-analysis by Meinel et al., the presence of the septal curvature was associated to all-cause mortality (*p* = 0.002), PE-related mortality (*p* = 0.0067), and adverse clinical outcomes (*p* = 0.0008) [28]. 

Lyhne et al. recently failed (*p* = 0.055) in demonstrating that the leftward curvature of the ventricular septum positively matched with more than twice the risk of clinical deterioration in patients with acute PE [23].

### 3.6. Left Atrial Size

The abrupt increase in pulmonary vascular resistance, which follows thromboembolic obstruction, can lead to RVD and failure. Subsequently, a decrease in pulmonary venous return with an insufficient filling of the LA may occur.

Several studies validated the use of LA sizes as a prognostic tool in patients with PE [16,29,30,31]. According to Guo et al., the left-to-right diameter and anterior-posterior diameter of LA showed a positive correlation with the prognosis of PE [31]. A reduced volume of LA was the best predictor of an adverse outcome, according to Aviram et al. [30]. In their study, they found a higher mortality rate among patients with an LA volume < 62 mL compared to those with an LA volume > 62 mL (19.6% vs. 8.9%, respectively; *p* < 0.001). Recently, in a study performed on 350 patients diagnosed with acute PE, the reduction in the volume of LA showed a positive correlation with adverse events (*p* ≤ 0.01) and death (*p* = 0.03) related to PE [30].

### 3.7. Pulmonary Artery Distensibility

Pulmonary artery distensibility (PAD) has proven to be a sensitive and specific marker for pulmonary hypertension [32]. Recently, the use of ECG-gated retrospective CTPA in the assessment of PAD has been investigated [33,34]. As reported by Liang et al., artery distensibility can be calculated using the following formula:

PAD = ([*Ss* − *Sd*]/*Sd*)/(*SBP* − *DBP*)

where *Ss* and *Sd* indicate the maximum and minimum luminal area, respectively; *SBP* defines the systolic blood pressure, and *DBP* indicates the diastolic blood pressure [35]. As suggested by Wang et al., measurements must be established on a section of the main PA, conventionally halfway between its origin and its bifurcation, adjusted to be perpendicular to the longitudinal axis in both the axial and coronal planes [36]. Furthermore, according to Wang et al., through PAD, the use of retrospective ECG-gated CTPA would allow providing information on cardiac function and assessing the stratification of APE risk [36].

### 3.8. Coronary Artery Calcifications

Coronary artery calcifications act as an important determinant of coronary artery disease [37]. Patients undergoing CT for suspected PE are more likely to have coronary artery calcifications [38]. Few studies in the literature have investigated the importance of coronary artery calcifications on both short- and long-term mortality in patients with APE [39,40]. Ng et al, in their study of 1023 patients diagnosed with PE, showed that long-term all-cause cardiovascular mortality was 2.2 higher than in patients without cardiovascular diseases [41]. Heidinger et al. found that severe or moderate coronary artery calcifications were associated with PE-related 30-day mortality of 9.5% (*p* = 0.049) and 6.7% (*p* = 0.033), respectively [40].

## 4. COVID-19 Pneumonia

PE is a common complication in patients with SARS-CoV-2 pneumonia [42]. The triggering cause of PE following an acute infection, especially in the respiratory tract, would be due to local inflammation causing the local activation of coagulation and vasoconstriction. Endothelial dysfunction, a state of hypercoagulation with activation of platelets and leukocytes, and later thrombin generation and widespread fibrin deposition, are related to the increased risk of thromboembolic events in SARS-CoV2 infection [43,44].

**Figure 4:**
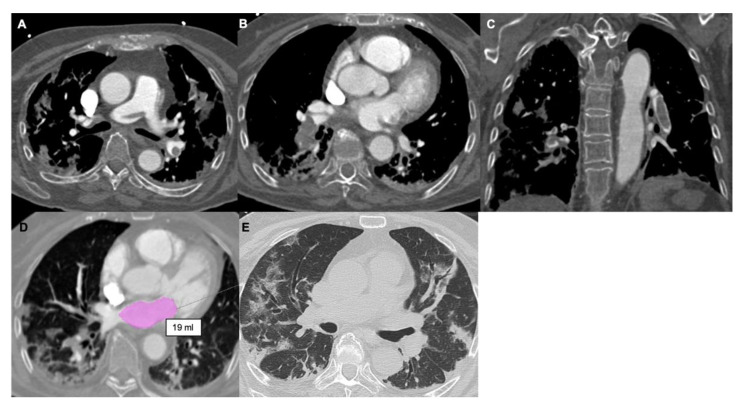
(**A**,**B**) Axial and coronal (**C**) contrast-enhanced CT scans show saddle embolism and multiple eccentric emboli involving the lower lobar branches of the pulmonary arteries. (**D**) Left atrium, purple. There is decreased LA volume, 19 mL. (**E**) Axial scans in the lung window show peripheral ground-glass opacities in association with consolidation areas involving both lungs.

Ippolito et al. observed that 76/170 (44.7%) patients with SARS-CoV-2 developed PE without having higher risk factors for venous thromboembolism [42]. In addition, PE-positive patients showed slightly longer hospitalization times than PE-negative patients, with a mortality rate among PE-positive patients of 54.2% at three months [42]. Moreover, in the study by Masselli et al., 26/60 (40%) patients with SARS-CoV-2 were found to be positive for PE at CTPA. In these patients, the probability of being admitted to the intensive care unit was higher than those who did not have PE (19/26 vs. 11/34 patients) and more frequently required mechanical ventilation (15/26 patients vs. 9/34 patients) [45] (Figure 4).

## 5. Dual Energy CT

According to recent studies, the use of new CT technologies such as dual-energy computed tomography (DECT) would provide additional information compared to single-energy CT with lower contrast doses and radiation [46,47,48]. Using monoenergetic image reconstructions, the attenuation of the vessel and the contrast–noise ratio can be improved while using a small amount of contrast agent. 

DECT would allow the simultaneous evaluation of the vascular and parenchymal distribution of iodine in the lungs, allowing the visualization of perfusion defects without an excessive dose of radiation [49,50,51]. According to some Authors, the detection of peripheral intrapulmonary clots would be improved by the addition of DECT to the CTPA. In their study, Weidman et al. showed that the DECT iodine map allows detecting small pulmonary emboli in 1% of patients that would otherwise be lost [46]. Several studies showed a positive correlation between perfusion defects, signs of RVD, and PA obstruction [52,53,54]. On the other hand, according to Monti et al., DECT did not yield additional diagnostic benefits in the detection of patients with APE compared to single CT energy [55].

More studies examined contrast medium volume reduction with DECT [56,57,58,59]. Reducing contrast medium volume with the application of DECT can help patients with impaired renal function, which is most commonly seen among those who have a high risk of PE [60]. However, also concerning CTPA, in recent years some strategies have been developed to optimize its technical parameters (e.g., reduced acquisition time, low-kVp protocols, high pitch imaging) [61,62]. 

Recent studies demonstrated the feasibility of performing CTPA using a minimal amount of iodinated contrast medium while maintaining sufficient image quality to exclude or diagnose PE [63,64]. 

**Figure 5:**
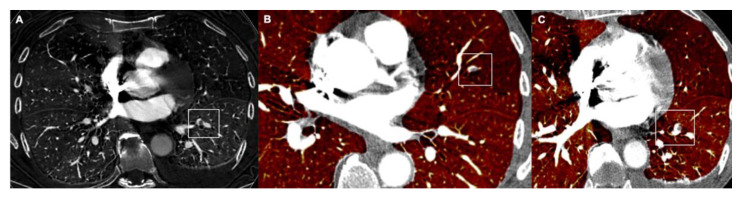
(**A**) The DECT iodine map allows for the detection of small pulmonary emboli in the left lower lobe. Simultaneous evaluation of the vascular and parenchymal distribution of iodine in the lungs with relief of a small embolus in the lingula (**B**) and the left lower lobe (**C**).

Silva et al., compared protocols performed with 20 or 40 mL contrast medium in patients with suspected PE [65]. In both protocols, vascular contrast enhancement was >250 HU, thus demonstrating that 20 mL contrast medium administration was sufficient for the evaluation of pulmonary arteries in patients with a clinical suspicion of APE [65] (Figure 5).

## 6. Conclusions

CTPA is a first-line tool for APE diagnosis that is also capable of quantifying PE severities with high accuracy, wide availability, and fast response times. Using a minimal amount of contrast medium (20 mL), it is still possible to maintain an image quality necessary to exclude or diagnose PE.

## Data Availability

Not applicable.

## Abbreviations

APE	Acute pulmonary embolism
CTOI	CT Obstruction Index
CTPA	Computed Tomography Pulmonary Angiography
IVC	Inferior vena cava
LA	Left atrium
LV	Left ventricle
PA	Pulmonary artery
PAD	Pulmonary artery distensibility
PE	Pulmonary embolism
RA	Right atrium
RV	Right ventricle
RVD	Right ventricular dysfunction
